# The relationship between the expression of soluble programmed cell death-1 and cancer pain: A protocol for systematic review and meta analysis

**DOI:** 10.1097/MD.0000000000025555

**Published:** 2021-04-30

**Authors:** Guangfeng Zhu, Yi Jiang, Hejie Wang, Shichao Shao

**Affiliations:** Department of Anesthesiology, Wenzhou Hospital of Integrated Traditional Chinese and Western Medicine,Wenzhou, Zhejiang province, China.

**Keywords:** Soluble programmed cell death-1, cancer pain, meta-analysis, protocol

## Abstract

**Background:**

The immune checkpoint programmed cell death-1 (PD-1) plays a critical role in immune regulation. Recent studies have demonstrated functional PD-1 expression in peripheral sensory neurons, which contributes to neuronal excitability, pain, and opioid analgesia. However, the relationship between the expression of soluble programmed cell death-1(sPD-1) and cancer pain is controversial. The purpose of this study was to evaluate the relationship between sPD-1 expression level and cancer pain through meta-analysis.

**Methods:**

Studies were selected from Pubmed, Web of science, Embase, Google Scholar, and Chinese National Knowledge Infrastructure, and the Chinese Biomedical Literature Database based on inclusion and exclusion criteria. The standard mean difference (SMD) and 95% confidence interval (CI) were calculated using the random-effect model or fixed-effect model to assess the association between sPD-1 expression level and cancer pain. All analyses were performed with the Stata 14 software.

**Results:**

This review will be disseminated in print by peer-review.

**Conclusion:**

The results of this study will help us to determine whether the expression level of sPD-1 is related to cancer pain.

**Ethics and dissemination:**

The private information from individuals will not be published. This systematic review also should not endanger participant rights. Ethical approval is not available. The results may be published in a peer-reviewed journal or disseminated in relevant conferences.

**OSF REGISTRATION NUMBER:**

DOI 10.17605/OSF.IO/WDPUY.

## 1 Introduction

Cancer is a major disease that has negative impacts on human survival and health.^[1]^ Cancer pain is a common symptom of advanced cancer, and can bring huge physical and mental burden to patients, thus seriously affecting their life quality.^[2,3]^ With the increase of in-depth studies on cancer pain, it is concluded that cancer pain has become an area of concern for clinicians. Epidemiological investigations proved that more than 50% of cancer patients experience cancer pain, and the incidence of cancer pain in patients with advanced cancer is as high as 80%. Cancer pain is usually moderate or severe, and 50 to 80% of the pain cannot be effectively controlled.^[4]^ The pain can damage body function and disease resistance and greatly affect the outcome of surgery, radiotherapy, chemotherapy and other cancer treatments. During its development, cancer may release pain factors, including vascular endothelial growth factor, nerve growth factor, protease, prostaglandin, endothelin and bradykinin, which may further aggravate cancer pain. However, most cancer patients have no pain in the early stages of the disease.^[5]^ Therefore, scholars believe that different pain-related factors may occur in different stages of cancer. Patients’ body may have painful and analgesic effects. These factors interact with each other to positively and negatively regulate patients’ sensitivity to pain.^[6]^

PD-1 and programmed death-ligand 1 (PD-L1) constitute PD-1 / PD-L1 signal transduction pathway, which inhibits the production of growth factors and cell proliferation, and play an important role in activating T cells and regulating immune response.^[7–9]^ In addition, PD-L1 suppresses T-cell-mediated immune response and helps tumor cells avoid being recognized and killed by the immune system.^[10,11]^ In healthy bodies, the activation of PD-1/PD-L1 signaling pathway reduces the damage to the immune response of surrounding tissues and prevents autoimmune diseases.^[12]^ On the contrary, activating this pathway can reduce the immune function of T cells in local tumor microenvironment, mediate tumor immune escape and promote cancer progression. Related studies have exhibited that the exogenous administration of PD-L1 can produce analgesic effects in normal mice, but blocking endogenous PD-L1 and PD-1 will cause pain.^[13]^ Primary nociceptors have some similarities with immune cells and can communicate with immune cells. The nociceptors express the main immunomodulators.^[14]^ sPD-1 is a blocker of PD-1/PD-L1 signal pathway and can inhibit the expression of PD-1^[15–17]^ and block the PD-L on tumor cells to promote tumor immunity, and it is closely related to the immune function of the body. Therefore, the abnormal expression of sPD-1 may be correlated with the occurrence and development of various diseases, including tumors. sPD-1 can be used to treat cancer pain. Therefore, more and more studies are focused on the relationship between sPD-1 and cancer pain.^[18,19]^

So far, no reliable evaluation system has been observed for the relationship between sPD-1 expression and cancer pain. Meanwhile, the relationship between the expression of sPD-1 and cancer pain is still not fully understood. Therefore, in order to clarify the relationship between the expression of sPD-1 and cancer pain, we systematically reviewed the relevant literature and summarized previous evidence on this topic.

## 2 Methods

### 2.1 Study registration

The protocol of the systematic review has been registered on Open Science Framework, and the registration number is DOI 10.17605/OSF.IO/WDPUY. This meta-analysis protocol is based on the Preferred Reporting Items for Systematic Reviews and meta-analysis Protocols (PRISMA-P) Statement Guidelines.^[20]^

### 2.2 Data sources and search strategy

The following electronic bibliographic databases are searched to identify relevant studies: Pubmed, Web of science, Embase, Google Scholar, and Chinese National Knowledge Infrastructure, and the Chinese Biomedical Literature, up to December 2020. The combination of Medical Subject Headings words and free words is adopted in the search. References of the literature are also included. According to different characteristics of the database, the retrieval strategy of title, abstract or keyword is adjusted. The languages are limited to Chinese and English. The search terms are illustrated in Table 1.

**Table 1:** Search strategy in PubMed database.

Number	Search terms
#1	Pain[MeSH]
#2	Suffering, Physical[Title/Abstract]
#3	Ache[Title/Abstract]
#4	Pain, Burning[Title/Abstract]
#5	Pain, Crushing[Title/Abstract]
#6	Pain, Migratory[Title/Abstract]
#7	Pain, Radiating[Title/Abstract]
#8	Pain, Splitting[Title/Abstract]
#9	Aches[Title/Abstract]
#10	Burning Pain[Title/Abstract]
#11	Burning Pains[Title/Abstract]
#12	Crushing Pain[Title/Abstract]
#13	Crushing Pains[Title/Abstract]
#14	Migratory Pain[Title/Abstract]
#15	Migratory Pains[Title/Abstract]
#16	Pains, Burning[Title/Abstract]
#17	Pains, Crushing[Title/Abstract]
#18	Pains, Migratory[Title/Abstract]
#19	Pains, Radiating[Title/Abstract]
#20	Pains, Splitting[Title/Abstract]
#21	Physical Suffering[Title/Abstract]
#22	Physical Sufferings[Title/Abstract]
#23	Radiating Pain[Title/Abstract]
#24	Radiating Pains[Title/Abstract]
#25	Splitting Pain[Title/Abstract]
#26	Splitting Pains[Title/Abstract]
#27	Sufferings, Physical[Title/Abstract]
#28	or/1-27
#29	Neoplasms[MeSH]
#30	Cancer[Title/Abstract]
#31	Tumors[Title/Abstract]
#32	Benign Neoplasms[Title/Abstract]
#33	Neoplasia[Title/Abstract]
#34	Neoplasm[Title/Abstract]
#35	Neoplasms, Benign[Title/Abstract]
#36	Benign Neoplasm[Title/Abstract]
#37	Cancers[Title/Abstract]
#38	Neoplasm, Benign[Title/Abstract]
#39	Tumor[Title/Abstract]
#40	or/29–-39
#41	Programmed death-1[Title/Abstract]
#42	PD-1[Title/Abstract]
#43	or/41-42
#44	#28 and #40 and #43

### 2.3 Inclusion criteria for study selection

The included articles must meet the following inclusion criteria:

(1) Studies assessing the association between the sPD-1 expression level and cancer pain.
(2) Patients diagnosed with cancer in the case group and healthy people in the control group.
(3) Detailed sPD-1 expression level data provided.
(4) Serial studies, including the latest study, from the same group of people were reported.
(5) The search was not limited to the language or date publication.

The criteria for excluding literature are summarized as follows:

(1) Non-human experiments were carried out.
(2) Repeatedly published literature.
(3) The lack of adequate information to calculate the statistical index standard mean difference (SMD).

### 2.4 Data collection and analysis

#### 2.4.1 Selection of studies

**Figure 1:**
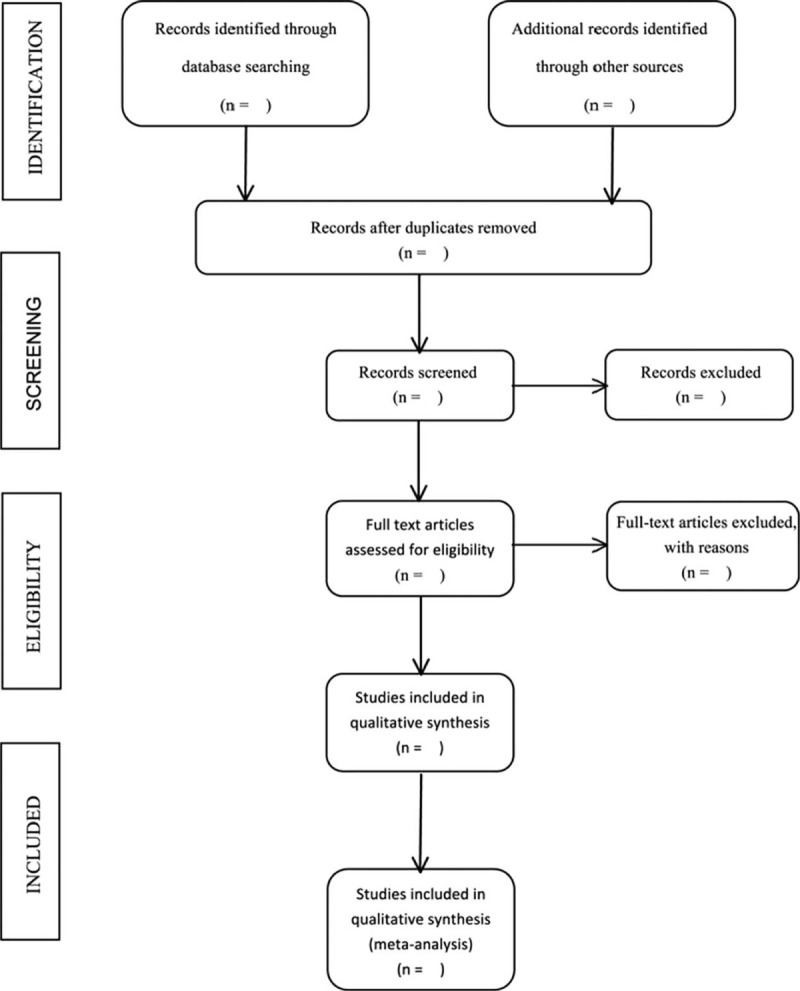
Flow chart of studies screening.

All reviewers received evidence-based training and adhered to the process that was summarized based on the PRISMA flowchart (Fig. 1). The two authors independently screened the literature on the basis of the title, abstract and key words of the literature, and excluded the irrelevant literature. The rest of the literature would be further confirmed by the two authors after reading the full text. The excluded research and the reasons for the exclusion were record. The existing dispute was settled by a third author.

#### 2.4.2 Data extraction and management

Two authors independently extracted the data from the eligible studies. Data entry was conducted with the EpiData software (version 3.0; The EpiData Association, Odense, Denmark). Disagreements were resolved by discussion with a third author or through consensus.

If the sPD-1 expression level data was not directly reported, all data were extracted from the statistical graph with Engauge Digitizer version 4.1 (Http://digitizer.sourceforge.net/). The following information was extracted: the first author, publishing year, country, ethnicity, sPD-1 and PD-1 positive expression, age, tumor type, specimen, detection method, sample size, etc.

### 2.5 Assessment of quality in included studies

The quality of all the included studies was evaluated by two authors independently based on the Newcastle–Ottawa scale (NOS) that was applied to evaluate the quality of observational studies.^[21–23]^ Disagreement were reported and resolved by a third author.

### 2.6 Measures of results

The data included in the study were continuous and standardized mean difference (SMD) and 95% confidence interval (CI) analysis.

### 2.7 Management of missing data

If there are insufficient or missing data in the literature, we would contact the author via email to request the data. If the data are not available, we would only analyze the currently available data and discuss its potential impacts.

### 2.8 Statistical analysis

Statistical analysis was performed with STATA 14.0 (STATA Corporation, College Station, TX). First, statistical heterogeneity tests were carried out on the included studies. If there is no statistical heterogeneity among the included literatures (I^2^ < 50%, *P* ≥ .1), a fixed effect model would be adopted. When there is statistical heterogeneity among the included literatures (*P* < .1, I^2^ > 50%), the sources of heterogeneity would be analyzed. Clinical heterogeneity would be treated by subgroup analysis. In the absence of significant clinical heterogeneity and methodological heterogeneity, statistical heterogeneity would be considered, and random effects models should be applied for analysis. If the clinical heterogeneity of the subgroup analysis is significantly higher, no meta-analysis would be performed, only a descriptive analysis.

### 2.9 Additional analysis

#### 2.9.1 Subgroup analysis

We conducted a subgroup analysis based on the sample type and detection method.

#### 2.9.2 Sensitivity analysis

The sensitivity analysis of each index was carried out by elimination method to check the stability of the results.

#### 2.9.3 Reporting bias

If the number of studies included in a certain outcome index is no less than 10, funnel chart will be used to evaluate publication bias.^[24,25]^

### 2.10 Ethics and dissemination

It is not applicable for this systematic review and meta-analysis to require an ethical approval, because this study is not involved in individual patient data. Besides, this review would be disseminated in peer-review journals.

## 3 Discussion

Cancer pain is a common complication of advanced cancer, and it can bring great burden to patients, thus seriously affecting their life quality.^[26–28]^ Whether PD-L1/PD-1 pathway is involved in the regulation of acute and chronic pain is a hot topic at present. PD-L1 decreased the excitability of neurons and increased the mechanical pain threshold.^[29–31]^ On the other hand, sPD-1 can neutralize PD-L1, increase the excitability of neurons and cause tactile pain.^[30]^ However, the relationship between the expression of sPD-1 and cancer pain still remains unclear, which does not take the advantages of the further development of future researches.

Therefore, there is an urgent need for a systematic review to the research on the relationship between the expression of sPD-1 and cancer pain. This paper forms our system review scheme, and describes the implementation of the review in details. The results of our review will be reported in strict accordance with PRISMA standards. By integrating previous literatures, this review objectively reveals the relationship between the expression of sPD-1 and cancer pain. The results of this upcoming study will help us to understand the relationship between the expression of sPD-1 and cancer pain, and will provide a reference to target new approaches for the treatment of cancer pain.

The advantages of this study include the following aspects. We included the latest literature. For the exploration of heterogeneity, we tried to avoid post-group subgroup analysis. In order to improve the credibility of the results, we conducted sensitivity analysis. Furthermore, we can relieve cancer pain through sPD-1 pathway so as to better make up for the deficiency of simple biotherapy.

In summary, this study will provide up-to-date evidence support for the relationship between sPD-1 expression and cancer pain, and provide a new strategy for the treatment of cancer.

## Author contributions

**Conceptualization:** Guangfeng Zhu.

**Data curation:** Guangfeng Zhu, Yi Jiang.

**Funding acquisition:** Guangfeng Zhu.

**Project administration:** Guangfeng Zhu.

**Supervision:** Guangfeng Zhu.

**Writing – original draft:** Guangfeng Zhu.

**Writing – review & editing:** Guangfeng Zhu, Yi Jiang.

**Resources:** Hejie Wang.

**Software:** Hejie Wang.

**Investigation:** Shichao Shao.

**Validation:** Shichao Shao.

**Visualization:** Shichao Shao.
